# Implementation of thyroid-related patient-reported outcomes in routine clinical practice

**DOI:** 10.3389/fendo.2022.1000682

**Published:** 2022-09-28

**Authors:** Per Karkov Cramon, Jakob Bue Bjorner, Mogens Groenvold, Victor Brun Boesen, Steen Joop Bonnema, Laszlo Hegedüs, Ulla Feldt-Rasmussen, Åse Krogh Rasmussen, Torquil Watt

**Affiliations:** ^1^ Department of Medical Endocrinology and Metabolism, Copenhagen University Hospital, Rigshospitalet, Copenhagen, Denmark; ^2^ Department of Clinical Physiology and Nuclear Medicine, Copenhagen University Hospital, Rigshospitalet, Copenhagen, Denmark; ^3^ Department of Public Health, University of Copenhagen, Copenhagen, Denmark; ^4^ QualityMetric Incorporated, LLC, Johnston, RI, United States; ^5^ Palliative Care Research Unit, Bispebjerg and Frederiksberg Hospital, Copenhagen, Denmark; ^6^ Department of Internal Medicine, Copenhagen University Hospital Herlev Gentofte, Copenhagen, Denmark; ^7^ Department of Endocrinology and Metabolism, Odense University Hospital, Odense, Denmark; ^8^ Department of Clinical Medicine, Faculty of Health and Clinical Sciences, University of Copenhagen, Copenhagen, Denmark

**Keywords:** patient-reported outcomes, quality of life, thyroid disease, thyroid dysfunction, routine, clinical practice, EMA

## Abstract

Patient-reported outcomes (PROs) are increasingly used in clinical practice to improve clinical care. Multiple studies show that systematic use of PROs can enhance communication with patients and improve patient satisfaction, symptom management and quality of life. Further, such data can be aggregated to examine health levels for patient groups, improve quality of care, and compare patient outcomes at the institutional, regional or national level. However, there are barriers and challenges that should be handled appropriately to achieve successful implementation of PROs in routine clinical practice. This paper briefly overviews thyroid-related PROs, describes unsolved quality of life issues in benign thyroid diseases, provides examples of routine collection of PROs, and summarizes key points facilitating successful implementation of thyroid-related PROs in routine clinical practice. Finally, the paper touches upon future directions of PRO research.

## Background

Quality of life (QoL) can be measured by patient-reported outcomes (PROs). According to the US Food and Drug Administration, “a PRO is a measurement of any aspect of a patient’s health status that comes directly from the patient (i.e., without the interpretation of the patient’s responses by a physician or anyone else)” (1). The prognosis of benign thyroid diseases, including hypothyroidism, is generally good with appropriate treatment, although many patients experience major QoL impairments at the time of disease presentation (2–6). Furthermore, despite good biochemical management and/or definitive treatment (e.g. goitre surgery), long-term follow-up studies have demonstrated persistent QoL deficits (3–5, 7, 8). Therefore, a major goal of clinical trials in thyroid diseases is to scrutinize new treatment strategies or novel therapies, with the aim of optimizing QoL. During the last two decades, PROs have been extensively and successfully applied in clinical studies of benign thyroid diseases. In the beginning, PROs were administered as paper surveys, but PROs are increasingly collected electronically *via* computers, tablets, or smart phones (9). The electronic methods of collecting PRO data allow real-time QoL measurements and presentation to clinicians in busy clinical practices, i.e. the electronic methods make routine PRO assessments feasible (10). However, no studies have described or systematically evaluated an implementation of PROs in routine thyroid clinical practice, despite accumulating evidence from other fields (e.g. oncology and inflammatory diseases) showing that routine use of PRO’s can improve clinical care (9, 11–14). The implementation of routine PROs is anticipated to improve several aspects of patient care by: identifying unrecognized and potentially treatable health problems, assessing the effectiveness of different treatments, detecting adverse effects, monitoring disease progress, improving patient-physician communication, and promoting shared decision making and patient empowerment (15–17). Eventually, these improvements may lead to better health outcomes in the form of reduced symptoms, better QoL and enhanced patient satisfaction (16), all known to be affected in individuals with thyroid disease in general, including hypothyroidism (18). Thyroid diseases are3interrelated both within the individual patient (e.g. co-existence3of hypothyroidism and goitre in classical Hashimoto’s disease) and across time (e.g. hypothyroidismresulting from treatment of non-toxic goitre or hypothyroidism leading to thyrotoxicosis in periods with overtreatment) and thus, implementation of PROs in an outpatient clinic should cover all benign thyroid conditions. Therefore, rather than focusing on hypothyroidism alone, this review considers such broader perspectives within a thyroid outpatient clinic.

## Thyroid-related PROs

There are two broad categories of PROs, i.e. disease-specific and generic. Disease-specific tools typically assess symptoms, functioning and patient perceptions that relate to a well-defined disease or condition, whereas generic tools evaluate broader categories that can be affected by a multitude of conditions. Disease-specific tools tend to be more sensitive to differences among clinically relevant groups and more responsive to small changes in health, as compared to generic tools (see **Table 1**) (23). The 36-item Short-Form Health Survey version 2 (SF-36v2) (21, 24, 25) and the EuroQol Group EQ-5D Health Survey (EQ-5D) (19) are two of the most widely used generic PRO instruments. In this paper, we focus on PROs developed for benign thyroid diseases. A systematic review from 2016 evaluated the measurement properties of PROs targeted for benign thyroid diseases (26). Based on measurement properties, that review recommended the Thyroid-Related Patient-Reported Outcome (ThyPRO) (22, 27–29) to assess QoL in patients with benign thyroid disease, while measurement properties for the Graves’ Ophthalmopathy Quality Of Life survey (GO-QOL) (20, 30–32) and the Thyroid Treatment Satisfaction Questionnaire (ThyTSQ) (33, 34) were deemed satisfactory for measuring QoL in Graves’ orbitopathy and treatment satisfaction with hypothyroidism, respectively. The strengths and limitations of all PROs intended for use in patients with benign thyroid diseases are tabulated in the systematic review paper by Wong et al. (26).

**Table 1:** There are two broad categories of PROs, generic and disease-specific.

	Generic PROs	Disease-specific PROs
**Purpose**	Intended for use in all individuals Overall impact of disease	Evaluation of symptoms, functioning and perceptions that relate to a well-defined disease or condition
**Population**	General population (healthy and non-healthy) Applicable across individuals with different diseases	Used for patients with a specific disease or condition
**Strengths**	Possible to compare QoL across populations or patient groups Enable comparisons between the relative burdens of diseases and their treatment benefits	Often more sensitive to differences among clinically relevant groups and more responsive to small changes in health Provide insights into the relationships between pathophysiology and QoL impairments
**Limitations**	Ceiling and floor effects are often more pronounced	Not possible to compare QoL across different groups of patients
**Examples**	SF-36v2 Health survey, EQ-5D	ThyPRO, GO-QOL

In this paper, we focus on PROs developed for benign thyroid diseases. EQ-5D, EuroQol Group EQ-5D Health Survey (19); GO-QOL, Graves’ Ophthalmopathy Quality Of Life survey (20); PROs, patient-reported outcomes; QoL, quality of life; SF-36v2, The 36-item Short-Form Health Survey version 2 (21); ThyPRO, Thyroid-Related Patient-Reported Outcome (22).

Factors to consider when choosing between generic and disease-specific PROs are shown in the table.

The ThyPRO survey covers all benign diseases (hypothyroidism, hyperthyroidism, goitre, and orbitopathy), which is advantageous for the reasons stated above. The ThyPRO survey is the most extensively validated PRO for benign thyroid diseases (26), and it has proven more responsive to clinical changes than the most widely used generic survey, i.e. the SF-36v2 Health Survey (21, 28). The original ThyPRO questionnaire consisted of 85 items summarized into 13 scales, as well as a single item measuring overall impact of thyroid disease on QoL. Later, the shorter 39-item version (ThyPRO-39) was developed, and it has demonstrated good measurement properties (35).

The GO-QOL survey consists of 16 items summarized in two subscales, and it is the most widely used PRO to assess QoL in Graves’ orbitopathy. Validated condition-specific PROs that may be relevant in a thyroid surgery setting exist, e.g. the Voice Handicap Index-10 (VHI-10) (36) or the Swallowing Quality of Life questionnaire (SWAL-QOL) (37), both of which have been applied to assess quality or treatment effects of thyroid surgery (38, 39).

The choice of PRO depends on the rationale for assessment, and hence researchers and clinicians should first decide what they want to measure. This might be e.g. impact of disease or its treatment on symptoms, daily activities, emotional well-being or side effects. The PRO selected should be well validated and have appropriate measurement properties for the disease in question. Finally, the researchers and clinicians need to identify the most appropriate tool for that task.

## Important QoL issues in benign thyroid diseases

Numerous studies of benign thyroid diseases have demonstrated deficits in multiple QoL domains (4–6, 40–43). In this section we discuss a few selected studies that have examined the impact of treatment on QoL. These studies also exemplify some of the many unsolved QoL issues in thyroid diseases. Stott et al. conducted a double-blind, randomized, placebo-controlled, parallel-group trial in 737 older adults with persistent subclinical hypothyroidism, where participants were randomized to levothyroxine or placebo (44). The results demonstrated that levothyroxine had no QoL benefits in older people with subclinical hypothyroidism, as measured by the ThyPRO survey. Shakir et al. conducted a randomized, double-blind, crossover study of 75 hypothyroid patients randomly allocated to 1 of 3 treatment arms, levothyroxine, levothyroxine + liothyronine or desiccated thyroid extract (45). Outcomes were similar among the three groups, as measured by four different validated PROs. However, patients most symptomatic on levothyroxine alone preferred and responded positively to therapy with levothyroxine + liothyronine or desiccated thyroid extract. Another recent study examined the effect of natural dessicated thyroid, as evaluated with ThyPRO-39 (35) and EQ-5D-L (19) at baseline and six months after initiation of treatment (46). Patients with levothyroxine unresponsive hypothyroidism experienced significant improvement in QoL. A recent consensus paper summarizes the evidence on levothyroxine/liothyronine combination therapy (47). However, whether to initiate substitution therapy in subclinical hypothyroidism and whether to choose combination therapy rather than monotherapy for QoL indications is currently unsettled which beyond QoL issues needs to take into consideration the time-dependent thyroid dysfunction related excess morbidity and mortality (48–50). For a comprehensive insight into various QoL issues in hypothyroidism we refer to a recent review (5).

Grove-Laugesen et al. randomized patients with first time diagnosis of Graves’ disease to vitamin D or placebo in addition to standard treatment with antithyroid drugs (42). Unexpectedly, the change of the ThyPRO Composite QoL scale was significantly worse in the Vitamin D group and the same tendency was seen for the Overall QoL-impact scale, Impaired Daily Life scale and Hyperthyroid Symptoms scale. Cramon et al. prospectively examined QoL before and 6 months after initiation of treatment in patients with toxic nodular goitre or Graves’ disease (51). At baseline, both patient groups had significantly poorer QoL on all ThyPRO and SF-36 domains, as compared with general population samples, and QoL improved markedly after six months of treatment for Graves’ disease while improving more modestly in patients with toxic nodular goitre. In both groups, QoL impairments persisted six months after treatment on multiple domains compared with general population samples. Törring et al. conducted a long-term follow-up study in patients with Graves’ disease treated with antithyroid drugs, radioiodine therapy, or surgery. The patients showed QoL impairments compared to general population norms 6-10 years after treatment (7), but patients treated with radioiodine therapy had worse long-term QoL than those treated with antithyroid drugs or surgery. Important limitations were lack of randomization and insufficient data on comorbidity.

As demonstrated by the above mentioned three studies, treatment may negatively and unexpectedly affect QoL, exemplified by QoL impairments both in the short- and long-term. Future clinical trials should be designed in such a way that both patients and clinicians can be advised to choose the most optimal treatment for effectively restoring the QoL.

## Examples of routine use of PROs

Implementing PROs in routine clinical practice is another option to better comprehend the impact of disease and treatment on QoL, and thereby better identify novel treatment targets. While no studies have been published concerning the potential effects of routine PRO assessment among thyroid patients, positive effects have been demonstrated in other patient groups. In a sentinel study by Velikova et al. 286 cancer patients were randomized to either 1) completion of PROs at routine clinical visits and feedback of results to physicians, 2) PRO completion, without feedback, or 3) no PRO assessment as a control group (10). Routine PRO assessment had a beneficial impact on physician-patient communication and resulted in better QoL and emotional functioning in some patients. Basch et al. assessed the impact of PRO monitoring in 766 cancer patients initiating chemotherapy (52). Participants were randomized to usual care or the PRO group, in which frequent symptoms associated with cancer treatment were self-reported at and between visits. Severity or worsening of symptoms triggered an email alert to a clinical nurse, and the reported symptoms were accessible for the treating oncologist as well. Integration of PROs significantly increased overall survival. Potential mechanisms were early responsiveness to symptoms preventing adverse downstream consequences and ability to tolerate chemotherapy longer than the usual care group. Short et al. assessed the value and feasibility of web-based PRO assessments within routine HIV care (53). The study included 1630 HIV patients who completed PROs in the outpatient clinic before their routine care consultation. The PROs effectively identified issues to address, particularly anxiety and suicidal ideation. The majority of patients (82%) and health care providers (82%) indicated that the PROs added value to the visits. Mistry and colleagues applied the Rheumatoid Arthritis Impact of Disease (RAID) survey at routine visits in routine care (54). The PROs could identify patients in remission or low disease activity with a high positive predictive value, which provided an opportunity to optimize outpatient visits for these patients. Additionally, the routine use of PRO data revealed a high burden of unmet needs, in particular fatigue and sleep problems, to be addressed in future clinical care.

Routine PRO assessment thus has the potential to improve physician-patient communication, improve QoL, identify unmet needs, potentially save outpatient visits in patients with better QoL, and for some diseases even improve overall survival. However, there are also barriers to implementation of routine PRO collection. Limited resources, both regarding funding as well as the time-consuming nature of developing electronic infrastructure and implementing routine PROs are important barriers (15). Clinicians can be reluctant to use PROs routinely because they fear it will add to their already busy workload, and further, the clinicians may contend that they already understand their patients’ problems and do not need extra patient-reported information (11). For more thorough summaries on the effect of routine PRO collection, including challenges and barriers, we refer to the review papers by Chen et al., 2013, Howell et al., 2015, and Nguyen et al., 2021 (12–14). It is unresolved whether these potential benefits apply to routine PRO collection in benign thyroid diseases.

## Implementation of thyroid-related PROs in routine clinical practice

There are several features of thyroid diseases, including hypothyroidism, that make routine PRO assessments particularly well suited for these patients: thyroid hormones affect all organ systems and thus cause a variety of different symptoms and QoL impairments including mental health issues; QoL impairments influence the choice of intervention (e.g. pressure symptoms and cosmetic concerns in non-toxic goitre); a substantial number of biochemically well-treated patients experience reduced QoL and may thus experience lack of congruence between the focus of the endocrinologist and themselves (55, 56); thyroid diseases are often chronic and occur at all ages, including during working life; thyroid diseases are rarely life-threatening, and focus on relevant QoL issues may thus have relatively large impact. The use of PROs in clinical studies of benign thyroid diseases is already well established. However, before the use of PROs can be extended to routine clinical practice, it is necessary to test the effect of routine PROs in feasibility studies and trials. Such studies and trials can be costly and require funding, which is often challenging for thyroid research.

Based on guidelines and studies of routine PRO collection, this section summarizes important topics to consider prior to implementation of routine thyroid-related PROs in feasibility studies and trials.

### Goals

There is a range of possible goals: investigating the impact of thyroid disease on QoL, evaluating treatment, identifying symptoms that may be alleviated, aiding in treatment decision-making, improving patient-physician communication, etc. Before implementing PROs in clinical practice it is important to clarify how the implementation is expected to have an effect, thereby guiding study design and selection of the most appropriate PROs (57).

### Selection of thyroid-related PROs

A literature search can both identify studies evaluating QoL and the PROs used for these measurements. An important consideration for the candidate tools is whether they address the symptoms and QoL domains most relevant for the target patient population (i.e. content validity). Next, it is crucial that the chosen PRO is thoroughly validated. In this respect, core considerations include: construct validity, reliability, responsiveness, discrimination ability, meaningful change, and translation validity (58). A systematic review from 2016 identified QoL instruments developed for thyroid diseases and graded these according to methodological quality and overall levels of evidence (26). The length of the PRO should be considered as the measure must neither be overly burdensome for patients nor for clinicians (4).

### Resources and education

Experience from previous studies shows that it is important not to underestimate the amount of time and human resources needed to establish and sustain PRO projects in clinical routine practice (15). Identifying local clinical key persons (e.g. physician or/and nurse) who can facilitate the administration of PROs and answer questions from clinicians and patients may be an important factor for successful implementation (9). The staff should be educated to administrate, understand, and evaluate the chosen PRO before the implementation starts. Moreover, technology and technical support should be in place to ensure efficient collection of electronic PRO data.

### PRO administration

The PROs should preferably be administered electronically, as paper surveys require time consuming data entry by the staff with risk of data entry errors. Collection *via* web-based systems accessible from the patients’ home with email prompts is one option. Another is a tablet solution in the out-patient clinic, where surveys are completed immediately before the consultation. Ideally, data should immediately be scored, and the results presented graphically in the electronic health record. In this way, the PRO results are readily available to the clinician to help guide the subsequent dialogue with the patient.

### Interpretability and feedback

**Figure 1:**
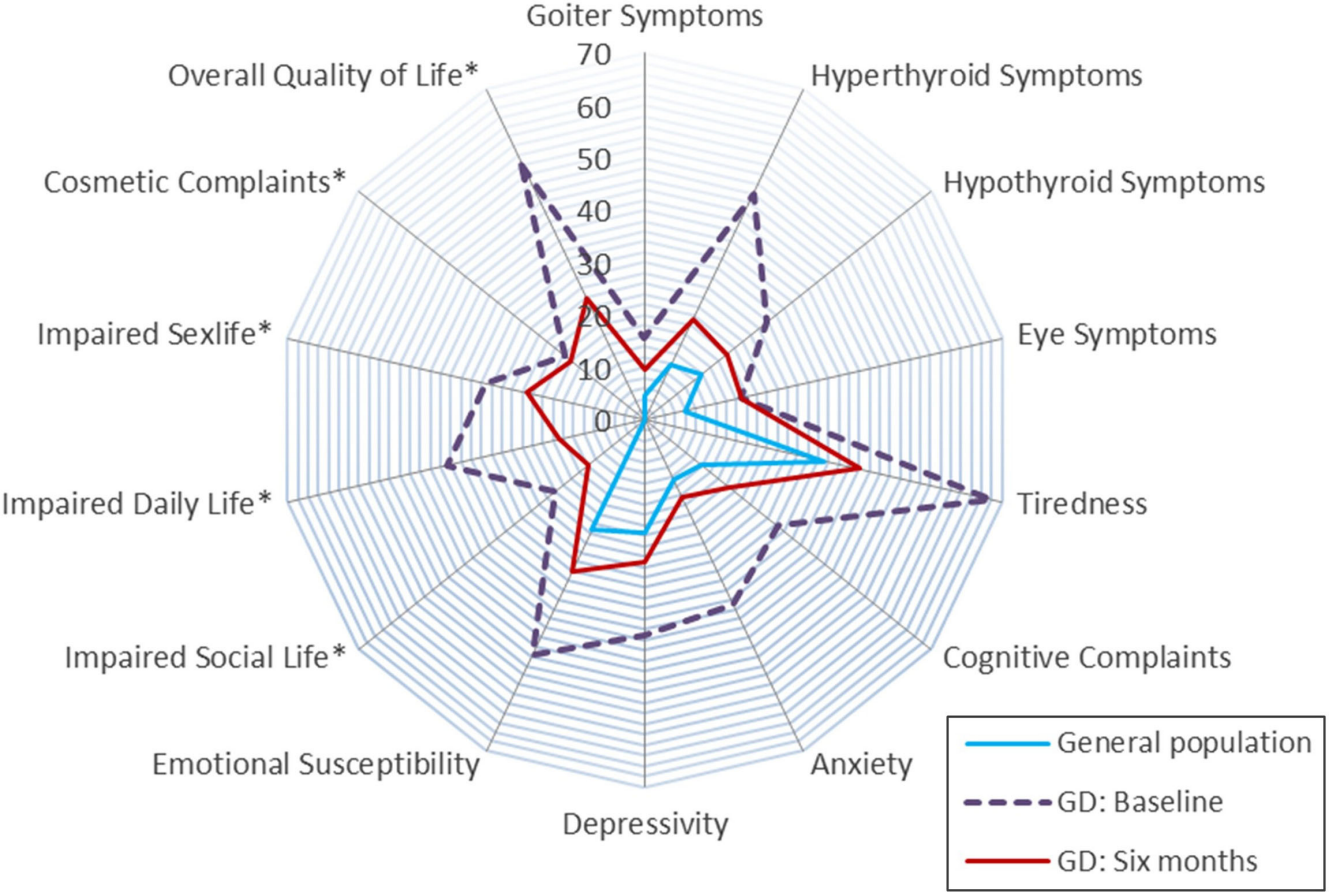
Radar plot showing ThyPRO scales scores for patients with Graves’ disease at baseline (i.e. before initiation of treatment, N=88) and 6 months after treatment (N=66), as well as scores from a general reference population (N=739). The scales range from 0 to 100, with higher scores indicating worse health status. *Items in these scales are asked with attribution to thyroid disease and cannot be answered by respondents from the general population. A radar plot gives a comprehensive overview of all quality of life domains and a few assessments can be shown in one plot. However, this type of graph is not well suited for multiple longitudinal assessments. The radar plot is based on data from Cramon et al. (51). GD: Graves’ disease.

**Figure 2:**
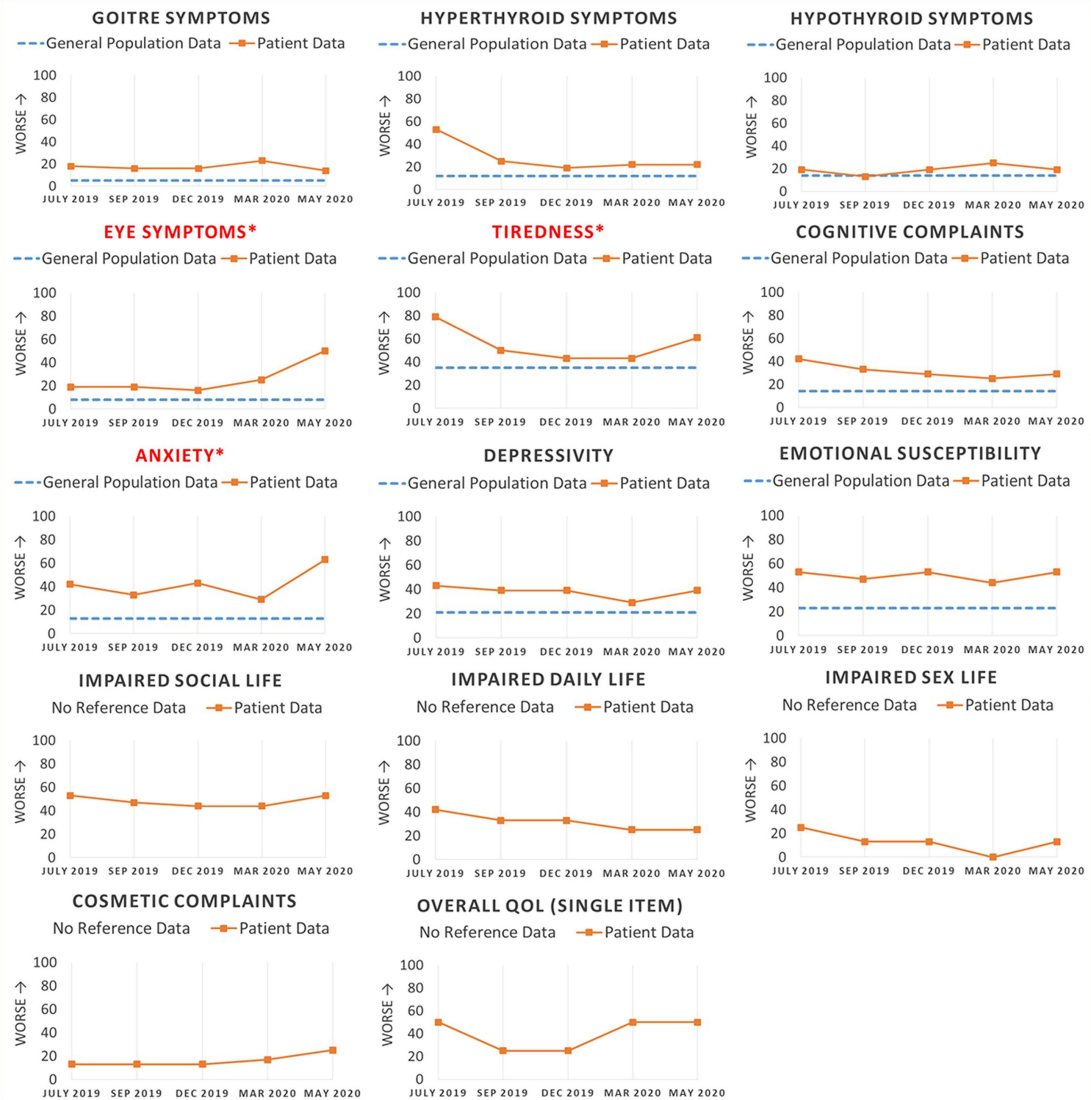
This figure illustrates how multiple longitudinal quality of life assessments can be presented to the clinician in the electronic health record. In this made-up example, a patient with newly diagnosed Graves’ disease completes the ThyPRO survey before each consultation. An indicator of worse QoL is seen to the left of the vertical axis as some PROs are scored opposite than ThyPRO (i.e. higher scores indicate better QoL). Reference scores are shown with blue dotted lines to facilitate the interpretation of patient scores. Scales are marked with an asterisk sign (*) and the scale name highlighted in red if the scores have deteriorated more than the MIC (minimal important change) (29), enabling the clinician to get a fast overview of domains with worsening QoL. Note: Items in scales of the two bottom rows are asked with attribution to thyroid disease and cannot be answered by respondents from the general population.

The PRO results should be easily understood and interpretable, for example by highlighting relevant changes and providing reference scores (see **Figures 1**, **2**). In case the PRO results are to be fed back to patients, they should also be interpretable to lay persons. However, the best way to convey PRO information to patients and clinicians has yet to be determined (59–62).

### Ability to act

Clinicians are less inclined to raise issues if they cannot do anything about them (17, 57, 63). This may explain why PRO implementation increases discussion of symptoms more often than of complex QoL issues (e.g. function or participation) (64). For example, the clinician may be more prone to initiate treatment for worsening of eye symptoms in Graves’ orbitopathy, as compared to handling daily function problems in a biochemically well-treated patient with hypothyroidism. However, recognizing that more complex QoL issues may be as important as more traditional measures might have great impact for the patient and can enhance treatment satisfaction. Additionally, poor or worsening QoL in euthyroid patients with Hashimoto’s thyroiditis can easily be attributed to thyroid disease despite it may be caused by other undiagnosed (autoimmune) diseases. Therefore, QoL deterioration may help the clinician consider non-thyroid disease or consider the possibility to change treatment strategy (e.g., switch to combination therapy). An automatic algorithm assessing PRO data collected between visits may identify patients in need of an extra consultation or vice versa, omission of a scheduled consultation in case of good QoL. Action guidance may be developed using a systematic and multidisciplinary approach (65–68).

### Evaluation of PRO value

A feasibility study with a limited number of patients and clinicians is recommended before more widespread implementation of routine PRO assessments. Such a study can identify challenges and barriers to PRO implementation, and moreover, examine how patients and clinicians value the PRO data. Ideally, feasibility studies should be followed by randomized controlled trials evaluating the impact of routine PRO collection on patient-physician communication, satisfaction, empowerment, QoL, and other health outcomes.

Previous studies of routine use of PRO have suggested that clinicians and researchers often experience practical challenges, such as administrative burden, problems with PRO interpretation, and perhaps even skepticism of the clinical value (15). We therefore recommend the abovementioned topics to be considered in the planning process in order to successfully implement PROs in routine clinical practice.

## Perspectives and future directions

The routine use of PROs in clinical practice has the potential to improve the clinical care of individual patients with thyroid diseases by improving physician-patient communication, improving QoL, identifying unmet needs, and perhaps saving unnecessary outpatient visits. Moreover, routine PRO assessments can provide data on the performance of different treatments in ‘real-world’ conditions (i.e. effectiveness), rather than under ideal or controlled circumstances (i.e. efficacy) (69). The use of routine PROs is steadily increasing in malignant and non-malignant diseases, both at individual centres and across entire health care systems (11). Routine PROs can be used for benchmarking and quality improvement purposes when implemented at multiple centres within a health care system. Importantly, results of PROs can guide patients in their choice of provider as well as the choice of treatment (11).

**Figure 3:**
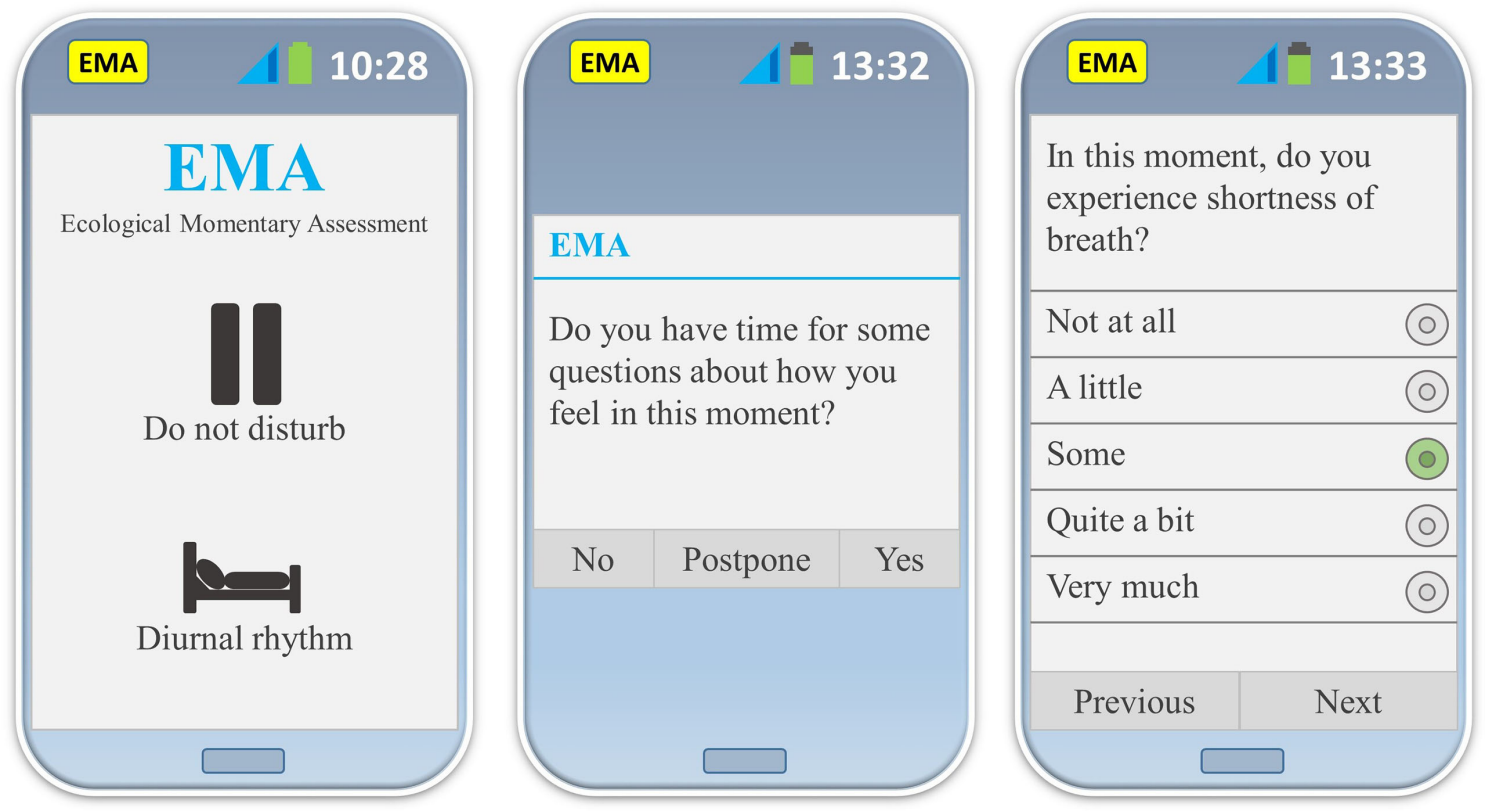
This figure illustrates three different user interfaces of the original smart phone app developed for EMA ThyPRO assessments (72). Left: it is possible to adapt the EMA assessments to the daily rhythm of the participants, and to pause EMA assessments (e.g. due to a business meeting). Middle: EMA notification. It is possible to postpone or decline EMA assessments on occasions where participants are unable to complete assessments. Right: EMA item with five response categories (in this example ‘Some’ has been chosen). It is possible to see as well as change all previous answers until the EMA assessment is completed. App: application; EMA: ecological momentary assessment.

In general, PROs are retrospective surveys, often with a recall period of one to four weeks. By ecological momentary assessments (EMAs) - an advancing method in the science of PROs - the current QoL is measured with repeated real-time assessments (70, 71). Typically, patients are notified several times daily to complete a very brief survey on their cell phone (see **Figure 3**). Theoretically, this approach has a number of advantages over retrospective surveys: 1) The assessments are not influenced by recall bias, 2) repeated daily samplings enable investigation of symptoms and QoL over time (e.g. daily fluctuations), 3) the method provides ecological validity, i.e. the assessments are made while patients are living their daily lives, which allows for contextual investigations of QoL, and 4) the data can be correlated to or even triggered by other relevant data retrieved by the cell phone (e.g. GPS-tracker, heart rhythm, diurnal rhythm, etc.) (72).

An EMA version of ThyPRO has recently been developed and applied in a few studies (71–74). The concept of EMA has the potential to increase our understanding of the impact of thyroid diseases and their treatment on QoL. EMA assessments in patients with hypothyroidism treated with liothyronine is an obvious example for application of this method, as the diurnal fluctuations of plasma thyroid hormones concentrations associated with this treatment may cause swift changes in symptoms. The EMA method could also be used for short periods to closer investigate symptoms of interest detected by routine retrospective PROs. When using PROs, there is a risk that the act of monitoring symptoms may affect symptom levels (so-called reactivity). The risk of reactivity may be higher with repeated real-time measurements for routine care. However, a previous study on momentary pain demonstrated only minimal reactivity (75). The conversion of retrospective PROs to momentary versions can be a difficult task as the interpretation of the momentary wording of the questions may be different from that of the retrospective wording. A study by Boesen et al. demonstrated that cognitive interviewing can be an efficient tool for developing and evaluating momentary questions (71).

There are important limitations in current QoL research in thyroid diseases. As already mentioned, it is well-documented that patients with benign thyroid diseases experience both short- and long-term QoL impairments. To what extent such deficits are due to thyroid disease per se, pre-existing morbidity or subsequent co-morbidity is currently unsettled. More research is needed to examine the relationship between QoL and the time-dependent thyroid dysfunction-related excess morbidity. Implementation of thyroid-related PROs in daily clinical practice could potentially contribute to expand our knowledge on this important topic. In general, PROs do not contain questions about patient preferences, although these preferences are very important in treatment decisions. In research studies, The PROs can be supplemented with questions about patient preferences when relevant for the research questions.

The movement towards patient-centered health care has resulted in a greater interest in symptoms and QoL issues reported directly by patients. The increasing use of PROs in clinical research has expanded our knowledge of the impact of thyroid diseases and their treatment on QoL. We suggest implementation of PROs in routine clinical practice to be established through meticulously designed studies and trials questing the optimal mode and evaluating its impact on key patient-centered outcomes as well as health service performance. Routine QoL assessments provide the opportunity to monitor the impact of treatment on the outcomes most meaningful for patients and to help shaping the delivery of patient-centered health care.

## Author contributions

PC participated in the planning process, wrote the manuscript draft, and approved the final manuscript. JB, MG, VB, SB, LH, UF-R, ÅR and TW participated in the planning process, read, and commented the manuscript draft, and approved the final manuscript. All authors contributed to the article and approved the submitted version.

## Funding

UF-R’s research salary was sponsored by a research grant from Kirsten and Freddy Johansen’s Foundation

## Conflict of interest

Author JB was employed by QualityMetric Incorporated, LLC.

The remaining authors declare that the research was conducted in the absence of any commercial or financial relationships that could be construed as a potential conflict of interest.

## Publisher’s note

All claims expressed in this article are solely those of the authors and do not necessarily represent those of their affiliated organizations, or those of the publisher, the editors and the reviewers. Any product that may be evaluated in this article, or claim that may be made by its manufacturer, is not guaranteed or endorsed by the publisher.
